# A case-control study on correlation between the single nucleotide polymorphism of *CLEC4E* and the susceptibility to tuberculosis among Han people in Western China

**DOI:** 10.1186/s12879-021-06448-2

**Published:** 2021-08-10

**Authors:** Wenjing Zhou, Lijuan Wu, Jiajia Song, Lin Jiao, Yi Zhou, Juan Zhou, Nian Wang, Tangyuheng Liu, Zhenzhen Zhao, Hao Bai, Tao Wu, Binwu Ying

**Affiliations:** Department of Laboratory Medicine, West China Hospital, Sichuan University, Chengdu, 610041 People’s Republic of China

**Keywords:** Tuberculosis, CLEC4E, Single nucleotide polymorphisms, Disease susceptibility

## Abstract

**Background:**

Tuberculosis (TB) is one of the leading causes of morbidity and mortality in Western China. Preclinical studies have suggested the protective effect of the C-type lectin receptor of family 4 member E (*CLEC4E*) from TB. Herein, we investigated the association between* CLEC4E* gene variants and TB susceptibility in a western Chinese Han population.

**Methods:**

We genotyped four single nucleotide polymorphisms (SNPs) rs10841856, rs10770847, rs10770855 and rs4480590 in the *CLEC4E* gene using the improved multiplex ligation detection reaction (iMLDR) assay in 900 TB cases and 1534 healthy controls.

**Results:**

After stratifying the whole data by sex, it was found that males exhibited mutant allele G of rs10841856 was more strongly associated with increased TB risk after Bonferroni correction (OR = 1.334, 95% CI: 1.142–1.560; *P* < 0.001 after adjusting for age; *p* = 0.001 after Bonferroni correction). The genetic model analysis found that rs10841856 was associated with the increased risk of TB among males under the dominant model (OR = 1.557, 95% CI = 1.228–1.984, *P* < 0.001 after adjusting for age, *P* < 0.001 after Bonferroni correction). Bioinformatics analysis suggested that rs10841856 might fall in putative functional regions and might be the expression quantitative trait loci (eQTL) for CLEC4E and long noncoding RNA RP11-561P12.5.

**Conclusions:**

Our study revealed that rs10841856 in the *CLEC4E* gene might be related to increased TB risk, especially the dominant genetic model among male Han individuals from Western China

**Supplementary Information:**

The online version contains supplementary material available at 10.1186/s12879-021-06448-2.

## Introduction

Tuberculosis (TB) is the leading cause of death among infectious diseases worldwide. China, which is the third highest-burdened country, accounted for 8.4% of the total global cases in 2019 [1]. The pathogen of tuberculosis is the *Mycobacterium tuberculosis* (MTB). Although approximately one-third of the people worldwide are infected with MTB, it is worth noting that only 3–10% of them eventually develop active clinical TB during their lifetime [2]. The occurrence or development of tuberculosis is determined by the complex interaction between three factors, the MTB strain itself, environmental, and host genetic factors [3–5]. Host genetics has been revealed to be important in determining disease progression and outcomes after MTB infection by many animal models studies, twin and family studies, as well as numerous case-control studies [6].

When MTB invades the host, it initially faces the innate immunity, which is modulated by the innate immunity genes. Pattern recognition receptors (PRRs) are key signaling molecules of the innate immune system that affect the initial identification of MTB [7]. Numerous studies have shown that genetic variation of the of PPRs, such as C-type lectin receptors (CLRs), Toll-like receptors (TLRs), RIG-I-like receptors (RLRs), and NOD-like receptors (NLRs) or their adapter protein-coding gene, is involved in modulating MTB-mediated immune responses and participate in determining the outcomes of MTB infection [8–10].

Macrophage-inducible C-type lectin (Mincle) is a newly described macrophage-inducible CLR, encoded by the C-type Lectin Receptor 4E (*CLEC4E*). Trehalose-6,6′-dimycolate (TDM), also known as cord factor, is the most abundant cell wall glycolipid of MTB that is important for the initial identification of MTB. It has been reported that Mincle could be considered as the mammalian receptor for TDM from MTB [11]. Lu et al found impaired production of interleukin-6 together with tissue necrosis factor in TDM-stimulated macrophages from Mincle^−/−^ mice exposed to *Malassezia spp* [12]. Moreover, in response to a TB vaccine containing trehalose-6,6′-dibehenate (TDB), a synthetic analog of TDM, Mincle has been shown to have a pivotal role in the generation of Th1/Th17 cell immune responses and granuloma formation [13]. These studies all suggested that Mincle has a significant role in recognition of mycobacteria.

So far, only two studies have investigated the relationship between the *CLEC4E* gene and TB susceptibility in humans [14, 15]. Yet, these two studies both had a small sample size, and their results were controversial. In order to evaluate the possible function of *CLEC4E* gene variants in TB, more studies need to be conducted. Moreover, such research has never been carried out among the western China population. Consequently, this relatively large-scale study was designed to investigate whether the single nucleotide polymorphisms (SNPs) in the *CLEC4E* gene were associated with susceptibility to TB in a Han population from Western China.

## Methods

### Subjects

Chinese Western Han individuals with TB recruited from West China Hospital of Sichuan University between January 2014 and February 2016 were enrolled. TB was diagnosed according to TB guidelines [16] based on their laboratory test results, clinical symptoms, and radiological examination. The inclusion criteria are typical symptoms and signs of tuberculosis and meet at least one of the following conditions: (1) smear positive for at least two separate clinical specimens and/or culture positive for MTB and/or examination positive for MTB nucleic acid (TB-DNA) (2) CT and other imaging examinations showed typical manifestations of active tuberculosis. (3) pathological diagnosis supports tuberculosis lesions. Patients suffer from immunodeficiency, autoimmune diseases, or other infectious diseases were excluded. Healthy controls were enrolled from the same population in the same period from the Physical Examination Center in West China Hospital of Sichuan University. They were all healthy according to normal laboratory test, physical examination and imaging examination. Individuals with TB history or non-Han population were excluded. Finally, 900 TB cases and 1534 healthy controls were enrolled.

The study protocol has been reviewed and approved by the Ethics Committee of West China Hospital of Sichuan University. Written informed consent was obtained from all participants before performing any study-related procedure.

### SNP selection and genotyping

Genetic variation information of the *CLEC4E* and intergenic regions of its upstream and downstream were obtained from the dbSNP database https://www.ncbi.nlm.nih.gov/SNP/. Haploview V4.2 was then employed to run the TagSNPs with a threshold of r^2^ greater than or equal to 0.8 from rescored SNPs. TagSNP with a minor allele frequency of (MAF) > 0.20 according to 1000 Genomes Project in East Asian population was selected.

Peripheral blood samples were collected from 2434 individuals and transferred to the biological specimen bank of resources of “Tuberculosis Researches” in the Department of Laboratory Medicine, West China Hospital, Sichuan University for preservation, and the demographic information of these individuals was gathered. Genomic DNA was extracted by the QIAamp® DNA Blood Mini Kit (Qiagen, Hilden, Germany). Improved multiplex ligation detection reaction (iMLDR) method (Genesky Biotechnologies Inc., Shanghai, China) [17] was used to genotype SNPs. 10% of samples were randomly selected for re-genotyping to check for concordance and the reproducibility of the genotyping was 100%.

### Statistical analysis

The Chi-square test, independent t-test, and Mann-Whitney U test were applied for general variables. All these calculations were performed by SPSS 20.0 (IBM, Chicago, USA). The Hardy-Weinberg equilibrium (HWE), differences of genotype distribution, and allele frequency of candidate SNPs between the TB group and control group or between age and sex subgroup were analyzed using PLINK 1.07 software [18]. Unconditional logistic regression models were used to test for the dominant model and recessive model. Data were adjusted for age and sex. Odds ratios (OR) with 95% confidence intervals (95% CI) and *P*-values were calculated. Haploview version 4.2 was used to examine the linkage disequilibrium (LD) by D′ and r^2^ value, haplotype structure and haplotype frequencies were estimated. A *P* value < 0.05 was considered statistically significant. Bonferroni correction was used to correct for multiple testing.

### Functional annotation

SNPs in strong LD (r^2^ > 0.90) with the SNPs associated with TB risk were identified according to the information from 1000 Genomes Project [19]. The DNAse, protein binding and transcription factor binding motifs were analyzed using HaploReg vesion4.1 [20]. Additionally, in order to identify whether these genes could provide more explanations for the associations observed in these SNPs, we used data from the GTEx project [21] so as to analyze if these variants have an effect on expression quantitative trait loci (eQTL). We searched the bioinformatics website lncRNASNP2 database [22] (http://bioinfo.life.hust.edu.cn/lncRNASNP/#!/) to obtain more information about long noncoding RNA (lncRNA).

## Results

### Characteristics of the study subjects

Finally, 900 TB patients and 1534 healthy Chinese Han individuals were enrolled in our study. The positive rate for TB-DNA results among patients was 50.5%, which was a little higher than those of MTB smear and culture (50.5% vs. 32.8 and 33.7%, respectively), as shown in a previous article published by our research group [23]. Compare with the control group, the ratio of male /female in the TB group was higher (1.151 vs. 1.514, *P* < 0.001). The median age was 41 (27, 57) years for the TB cases and 36 (29, 45) years for the controls (*P* < 0.001); details are shown in Table 1.

**Table 1:** Basic characteristics of the participants enrolled in the study

Characteristic	TB, n (%)	Control, n (%)	*P*-value
**Sex**			
Male	542 (60.22)	821 (53.52)	**<0.001**
Female	358 (39.78)	713 (46.48)	
Male/Female ratio	1.514	1.151	
**Age (years)**			
Median	41 (27,57)	36 (29,45)	**<0.001**
Male, Median	44.5 (26,58)	38 (30,47)	**<0.001**
Female, Median	37 (27,54)	34 (29,43)	**<0.001**
**Age group**			
< 40	417 (46.33)	922 (60.10)	**<0.001**
≥ 40	483 (53.67)	612 (39.90)	
**Age group-sex**			**0.036**
< 40			
Male	228 (54.67)	447 (48.48)	
Female	189 (45.32)	475 (51.52)	
≥40			0.185
Male	314 (65.01)	374 (61.11)	
Female	169 (34.99)	238 (38.89)	

Data are present as Median (25%quartile,75 quartile) or n(%)

### SNPs of *CLEC4E* are associated with susceptibility to TB

**Fig. 1:**
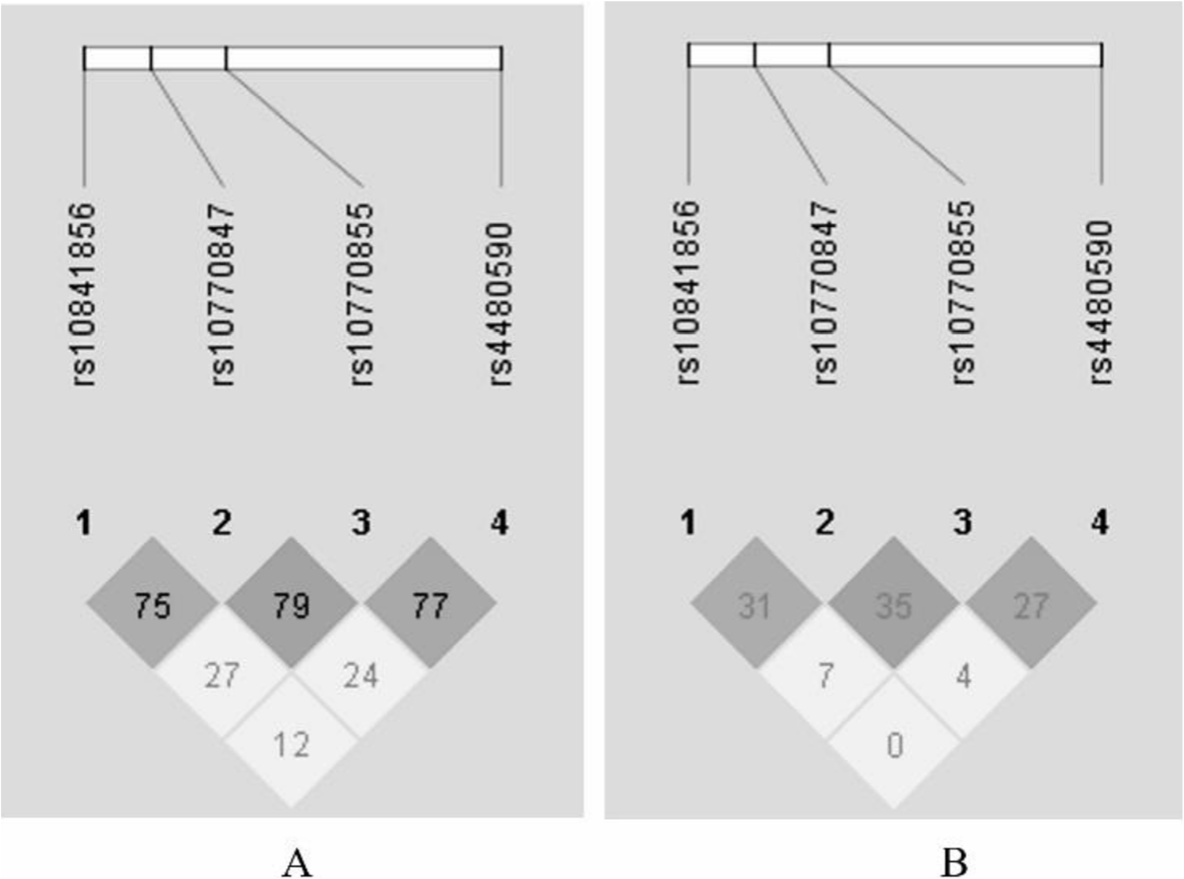
Linkage disequilibrium plot in D′ demonstrating adjacent strength between SNP pairs in the CLEC4E gene. D′ (**A**) and r^2^ (**B**) values were multiplied by 100. In (**A**), squares without a number have a value of 100, equal to a D′ value of 1. When two SNPs are completely linked, the D′ value is 1. In (**B**), squares without a number have a value of 80, equal to an r^2^ value of 0.8. The r^2^ values ≥0.8 were considered significant. The four SNPs in our study were not in linkage disequilibrium

Four SNPs of *CLEC4E* (rs10841856, rs10770847, rs10770855, rs4480590) were chosen for genotyping. Genotype frequencies for these four SNPs were all in Hardy-Weinberg equilibrium (*P* > 0.05). All of these four SNPs had a frequency of variants > 0.20 and were included for further analysis. The chromosomal locations, functional annotations, *p*-values for the HWE test in control subjects, and MAFs of these candidate SNPs are summarized in Table 2. We performed haplotype analyses for all four variants in/near the *CLEC4E* gene. The LD patterns of these four *CLEC4E* SNPs are shown in Fig. 1; no haploblock was identified.

**Table 2:** Characteristics of *CLEC4E* SNPs

SNP	Chr: position	Functional	HWE-*P-value*	MAF
rs10841856	12:8692843	Intronic variant	0.834	0.430
rs10770847	12:8698471	Intergenic variant	0.664	0.298
rs10770855	12:8704521	Intergenic variant	0.294	0.426
rs4480590	12:8726911	Intergenic variant	0.379	0.252

SNP = single-nucleotide polymorphism. Chr = chromosome; HWE-*P* = *P* value of Hardy-Weinberg equilibrium tests in the control group; MAF = minor allele frequency;

### SNPs of CLEC4E depended on sex and age

A weak correlation was identified for the mutant G allele and GG and GA genotype of rs10841856 and the susceptibility of TB before Bonferroni correction (Table S1). Likewise, a weak correlation was also observed between rs10841856 and the risk of TB in the dominant model; nonetheless, statistical significance was lost after Bonferroni correction (Table S2).

When the whole data were stratified according to sex, the mutant G allele frequency of rs10841856 among male TB subjects (47.69%) was higher than among male controls (40.61%). Also, the mutant G allele was strongly associated with TB risk, with an adjusted OR of 1.334 (95% CI: 1.142–1.560; *P*<0.001 after adjusting for age; *P* = 0.001 after Bonferroni correction). It was observed that the homozygous mutant GG (21.03% vs. 16.32%, *P* = 0.002, *P* = 0.008 after Bonferroni correction) and heterozygous AG (53.32% vs. 48.72%, *P* = 0.002, *P* = 0.008 after Bonferroni correction) were more common in the TB group than in the control group. Likewise, rs10841856 was significantly associated with TB susceptibility with an adjusted OR of 1.557 (95% CI = 1.222–1.984; *P* < 0.001 after adjusting for age; *P* < 0.001 after Bonferroni correction) in the dominant model, whereas, no significant differences of rs10841856 were found between female TB subjects and female controls. These results suggested that allele G of rs10841856 might be a risk factor in TB subjects, especially in males. Meanwhile, a weak association between rs10770847 G allele (*P* = 0.044 after adjusting for age) and the risk of TB was found among males; however, after Bonferroni’s correction for multiple testing, both associations lost statistical significance (Table 3 and Table 4).

**Table 3:** Comparison of *CLEC4E* SNPs polymorphisms in relation to TB risk in Chinese Han population stratified by sex

SNP/Sex	Allele	Case n(%)	Control n(%)	OR (95% CI) ^*a*^	P*	*P*^*a*^	P***	*Genotype*	Case n(%)	Control n(%)	P*	P***
rs10841856/Male	G	517(47.69)	652(40.61)	1.334(1.142,1.560)	**<0.001**	**<0.001**	**0.001**	GG	114(21.03)	134(16.32)	**<0.001**	**<0.001**
A > G	A	567(52.31)	974(59.39)					AG	289(53.32)	400(48.72)	**0.002**	**0.008**
								AA	139(25.65)	287(34.96)		
rs10841856/Female	G	297(41.48)	606(43.06)	0.944(0.785,1.134)	0.486	0.537	1.000	GG	62(17.32)	132(18.51)	0.509	1.000
A > G	A	419(58.52)	769(56.94)					AG	173(48.32)	350(49.09)	0.608	1.000
								AA	123(34.36)	231(32.40)		
rs10770847/Male	G	355(32.75)	471(28.75)	1.188(1.005,1.405)	**0.027**	**0.044**	0.106	GG	54(9.96)	57(6.96)	**0.024**	0.096
A > G	A	729(67.25)	1167(71.25)					AG	247(45.57)	357(43.59)	0.194	0.776
								AA	241(44.46)	405(49.45)		
rs10770847/Female	G	203(28.43)	417(29.28)	0.973(0.795,1.189)	0.682	0.787	1.000	GG	28(7.84)	68(9.55)	0.416	1.000
A > G	A	511(71.57)	1007(70.72)					AG	147(41.18)	281(39.47)	0.756	1.000
								AA	182(50.98)	363(50.98)		
rs1077855 /Male	A	466(43.07)	686(41.83)	1.048(0.896,1.226)	0.522	0.556	1.000	AA	96(17.74)	134(16.34)	0.389	1.000
G > A	G	616(56.93)	954(58.17)					AG	274(50.65)	418(50.98)	0.616	1.000
								GG	171(31.61)	268(32.68)		
rs1077855 /Female	A	317(44.27)	600(42.31)	1.091(0.908,1.311)	0.387	0.352	1.000	AA	70(19.55)	126(17.77)	0.484	1.000
G > A	G	399(55.73)	818(57.69)					AG	177(49.44)	348(49.08)	0.829	q.000
								GG	111(31.61)	235(33.15)		
rs4480590/ Male	A	269(24.86)	429(26.25)	0.911(0.762,1.089)	0.416	0.307	1.000	AA	35(6.47)	50(6.12)	0.996	1.000
G > A	G	813(75.14)	1205(73.75)					AG	199(36.78)	329(40.27)	0.206	1.000
								GG	307(56.75)	438(53.61)		
rs4480590 /Female	A	178(24.93)	344(24.23)	1.089(0.881,1.346)	0.729	0.430	1.000	AA	25(7.00)	41(5.77)	0.464	1.000
G > A	G	536(75.07)	1076(75.77)					AG	128(35.85)	262(36.90)	0.852	1.000
								GG	204(57.14)	407(57.32)		

*P* *was calculated by the Chi-square test. P** and OR (95% CI) ^*a*^ was adjusted by age. P*** was calculated after Bonferroni correction. SNP = single-nucleotide polymorphism; OR = odds ratio; CI = confidence interval

**Table 4:** Comparison of *CLEC4E* SNPs in relation to TB risk in the Chinese Han stratified by sex (dominant and recessive model)

SNP/Sex	Dominant Model				Recessive Model			
	OR (95% CI) ^*a*^	P*	*P*^*a*^	P***	OR (95% CI) ^*a*^	P*	*P*^*a*^	P***
rs10841856/Male	1.557(1.222,1.984)	**<0.001**	**<0.001**	**0.001**	1.383(1.044,1.830)	**0.024**	**0.024**	0.096
rs10841856/Female	0.897(0.683,1.178)	0.520	0.436	1.000	0.971(0.693,1.360)	0.632	0.864	1.000
rs10770847/Male	1.194(0.958,1.488)	0.072	0.114	0.286	1.465(0.988,2.172)	0.049	0.057	0.196
rs10770847/Female	1.013(0.783,1.311)	0.999	0.920	1.000	0.831(0.522,1.322)	0.358	0.434	1.000
rs1077855/Male	1.046(0.827,1.323)	0.678	0.707	1.000	1.098(0.821,1.469)	0.499	0.528	1.000
rs1077855/Female	1.116(0.846,1.471)	0.481	0.439	1.000	1.133(0.816,1.573)	0.478	0.457	1.000
rs4480590/Male	0.859(0.688,1.071)	0.256	0.177	1.000	1.038(0.662,1.630)	0.795	0.870	1.000
rs4480590/Female	1.071 (0.824,1.391)	0.955	0.609	1.000	1.283(0.762,2.161)	0.433	0.349	1.000

*P* *was calculated by Chi-square test

*P*^a^ and OR (95% CI) ^a^ was adjusted by age

P *** was calculated after Bonferroni correction

SNP = single-nucleotide polymorphism; OR = odds ratio; CI = confidence interval

Next, we stratified the whole data according to age, which showed no significant differences between TB subjects and control in the < 40 years age group or between TB subjects and control in the ≥40 years age group. There were also no statistically significant differences between the allele frequencies and genotype distribution of the other 3 loci (rs10770847, rs4480590, and rs10770855) in TB patients and healthy controls before or after the whole data stratification in relation to sex/age after Bonferroni’s correction (Table S3 and S4).

### Functional annotation

Rs10841856 is an intronic region of *CLEC4E*. Using LD information from the 1000 Genomes Project, eight SNPs were strongly linked (r^2^ > 0.90) with rs10841856. Among them, rs11046135 was near the 5’UTR region of *CLEC4E*; rs7485954 was in the upstream transcript region, and the remaining six SNPs were located in intronic regions of the *CLEC4E* gene. Based on the data from the Encyclopedia of DNA Elements (ENCODE) project [24], rs7307228, rs4242896, rs7139227, rs11046135, and rs7485954 might fall in a strong promoter or/and enhancer activity region; rs10841847, rs7139227, rs10841856, and rs11046135 in a DNAse hypersensitivity site region; rs7139227 in a transcription factor binding region; rs10841847, rs7307228, rs6487242, rs7139227, rs4562874, rs10841856, rs11046135, and rs7485954 in the regulatory motif (Table S5). According to the GTEx project, these eight SNPs are expression quantitative trait loci (eQTLs) for *CLEC4E* and *RP11-561P12.5* and are associated with a decrease in *CLEC4E* and an increase in *RP11-561P12.5* (Table S6).

## Discussion

The role of host’s genetic factor in tuberculosis susceptibility has gained increasing attention in TB research over recent years. Mincle is an indispensable receptor for TDM-induced innate immune responses (such as granuloma genesis) and in vitro macrophage activation during mycobacterial infection [25]. In the present study of the Western Han Chinese population, rs10841856 minor G allele of *CLEC4E*, which was the coding gene of Mincle, significantly increased the susceptibility to tuberculosis, especially among male subjects. Interestingly, Deo et al [14] suggested that for rs10841847, the minor G allele was a risk factor of pulmonary tuberculosis infection in a northern Chinese population. According to the 1000 Genomes Project, rs10841847, which is also an intronic variation of *CLEC4E,* is in strong linkage disequilibrium (LD) with rs10841856 (D′ = 0.95). Our findings on the association with TB risk of rs10841856 in male individuals supported the suggestion of the involvement of *CLEC4E* genetic polymorphism in TB. Nevertheless, Bowker et al [15] genotyped four tagSNPs of *CLEC4E*, reporting no differences in these SNPs between South Africa TB patients and controls. Such different observations might reflect the existence of many confounding factors, including ethnic background and sample size.

TB has a higher incidence in males than in females. In 2018, males accounted for 68%, while females accounted for only 31% of TB patients in China [1]. In our study, TB was also more common in males than in female individuals. Recently, Haiko et al [26] conducted a Genome-Wide association study that emphasizes the importance of sex-stratification analysis, because strong sex-specific effects are found on both autosomes and X chromosomes, and these effects should be considered when studying the association with SNPs and TB. When the whole data were stratified according to sex, in rs10841856, the G allele was a risk genotype for TB, especially in males. A significant difference was also found only in males when the association was calculated under the dominant model. This study showed the impact of sex on TB for *CLEC4E* rs10841856. Sex-specific effects of gene SNPs have been previously described in some diseases, including TB [27–29] As far as we know, this is the first report that described sex-specific interactions for variants in *CLEC4E,* which could be used as a basis for replication studies in independent populations.

The rs10841856 polymorphism is located in the intronic region. Although genetic polymorphisms in intron regions are not generally thought to cause changes in the encoded amino acids, they may affect splicing, transcription, and expression of genes [30–32]. According to data from the GTEx project, rs10841856 might be an eQTL of *CLEC4E* and *RP11-561P12.5*. Rs10841856 polymorphism decreased the expression of *CLEC4E* and increased the expression of *RP11-561P12.5* in whole blood. The decreased expression of *CLEC4E* was associated with bacterial infection and has been observed in several studies [33, 34]. For MTB infection, Pahari et al [35]. observed that* CLEC4E *agonist could improve host immunity and reduced bacterial load in the lungs of the infected mice. They elucidated the novel role of *CLEC4E* in inducing autophagy during defending MTB infection. Rs10841856 might be associated with *CLEC4E* expression decrease, which may weaken the defense ability against MTB. *RP11-561P12.5* is a lncRNA located at chromosome 12: 8700957-8720209, adjoining *CLEC4E*. Although there are scarce reports on the biological functions of *RP11-561P12.5*, according to the lncRNASNP2 database, *RP11-561P12.5* may bind to miR-197-3p. *Van Rensburg* et al [36] demonstrated that the neutrophil-associated miR-197-3p showed significantly lower transcript levels in TB cases; meanwhile, miR-197-3p acted as a binding site on the 3’UTR region of IL-22 receptor IL22RA1, thereby affecting the production of IL-22 [37]. IL-22 can inhibit MTB growth within macrophages [38] and promotes the innate immune responses, thereby limiting damage during pathogen infections [39]. The rs10841856 polymorphism influences the expression of *RP11-561P12.5*. We speculated that by binding to miR-197-3p, lncRNA *RP11-561P12.5* might have a similar mechanistic effect on the production of IL-22 that are also involved in the occurrence of TB*.*

No association of the other 3 SNPs (rs4480590, rs10770847, and rs10770855) was found with tuberculosis in this study after Bonferroni correction. To date, there was no TB-related research on rs4480590, rs10770847 and rs10770855. These three SNPs may not be related to TB risk in the Western China Han population. However, multicenter studies with large samples are needed to further verify these findings.

The present study has some limitations. Firstly, SNPs were mainly detected in the intrinsic region. Thus, variants in exons and regulatory genetic sequences should be taken into consideration, which means that more comprehensive and systematic variants of association studies are needed in the future. Secondly, the individuals involved in our study were all from the Western China Han population, which suggests that as same as for any novel genetic association, our findings should be replicated in other population and functional tests, and pathway analyses are required to validate our findings further.

In conclusion, the strong association was observed between the G allele and the dominate model of rs10841856 and the susceptibility of TB among males in a western Chinese Han population. Rs10841856 and its strong LD SNPs are associated with a decrease in *CLEC4E* and an increase in *RP11-561P12.5*. Accordingly, rs10841856 in *CLEC4E* might be a novel mutation that has a significant role in increasing the risk of TB among the male Han population from Western China.

## Supplementary Information

**Additional file 1.Table S1.** Allele and genotype distributions of CLEC4E SNPs polymorphisms in the TB group and healthy controls. **Table S2.** Comparison of 4 SNPs in relation to TB risk in the Chinese Han population. **Table S3.** Comparison of 4 SNPs polymorphisms in relation to TB risk in Western Chinese Han population stratified by age. **Table S4.** Comparison of 4 SNPs in relation to TB risk in the Chinese Han population stratified. **Table S5.** Functional annotation of rs10841856 and its closely linked SNPs (r2>0.90) using data from the Encyclopedia of DNA Elements Project. **Table S6.** Analyses of expression quantitative trait locus (eQTL) in rs10841856 and its closely linked SNPs (r2>0.90). **Supplementary file 1.** STROBE Statement—Checklist of items that should be included in reports of case-control studies.

## Data Availability

The data and materials supporting the conclusions of the study are available from the corresponding author on reasonable request.

## Abbreviations

CLEC4E: C-type lectin receptor of family 4 member E
TB: Tuberculosis
SNPs: Single-nucleotide polymorphisms
IMLDR: Improved multiplex ligation detection reaction
OR: Odds ratio
CI: Confidence intervals
eQTL: Expression quantitative trait loci
MTB: *Mycobacterium tuberculosis*
PRRs: Pattern recognition receptors
CLRs: C-type lectin receptors
TLRs: Toll-like receptors
RLRs: RIG-I-like receptors
NLRs: NOD-like receptors
TDM: Trehalose-6,6′-dimycolate
TB-DNA: MTB nucleic acid
CT: Computed Tomography
MAF: Minor allele frequency
HWE: Hardy-Weinberg equilibrium
LD: Linkage disequilibrium
GTEx: Genotype-Tissue Expression
ENCODE: Encyclopedia of DNA Elements

## References

[1] Organization WH. Global Tuberculosis Report 2020. Available from https://apps.who.int/iris/bitstream/handle/10665/336069/9789240013131-eng.pdf

[2] O'Garra A, Redford PS, McNab FW, Bloom CI, Wilkinson RJ, Berry MP (2013). The immune response in tuberculosis. Annu Rev Immunol.

[3] Caws M, Thwaites G, Dunstan S, Hawn TR, Lan NTN, Thuong NTT (2008). The influence of host and bacterial genotype on the development of disseminated disease with mycobacterium tuberculosis. PLoS Pathog.

[4] Walzl G, Ronacher K, Hanekom W, Scriba TJ, Zumla A (2011). Immunological biomarkers of tuberculosis. Nat Rev Immunol.

[5] Gagneux S (2012). Host-pathogen coevolution in human tuberculosis. Philos Trans R Soc Lond Ser B Biol Sci.

[6] Orlova M, Schurr E (2017). Human Genomics of *Mycobacterium tuberculosis* Infection and Disease. Curr Genet Med Re.

[7] Mishra A, Akhtar S, Jagannath C, Khan A (2017). Pattern recognition receptors and coordinated cellular pathways involved in tuberculosis immunopathogenesis: emerging concepts and perspectives. Mol Immunol.

[8] Wevers BA, Geijtenbeek TB, Gringhuis SI (2013). C-type lectin receptors orchestrate antifungal immunity. Future Microbiol.

[9] Takeuchi O, Akira S (2010). Pattern recognition receptors and inflammation. Cell.

[10] Song J, Liu T, Jiao L, Zhao Z, Hu X (2019). RIPK2 polymorphisms and susceptibility to tuberculosis in a Western Chinese Han population. Infect Genet Evol.

[11] Matsunaga I, Moody DB (2009). Mincle is a long sought receptor for mycobacterial cord factor. J Exp Med.

[12] Lu X, Nagata M, Yamasaki S (2018). Mincle: 20 years of a versatile sensor of insults. Int Immunol.

[13] Richardson MB, Williams SJ (2014). MCL and Mincle: C-type lectin receptors that sense damaged self and pathogen-associated molecular patterns. Front Immunol.

[14] Kabuye D, Chu Y, Lao W, Jin G, Kang H (2019). Association between CLEC4E gene polymorphism of mincle and pulmonary tuberculosis infection in a northern Chinese population. Gene..

[15] Bowker N, Salie M, Schurz H, van Helden PD, Kinnear CJ, Hoal EG, Möller M (2016). Polymorphisms in the pattern recognition receptor Mincle gene (CLEC4E) and association with tuberculosis. Lung..

[16] Lewinsohn DM, Leonard MK, LoBue PA, Cohn DL, Daley CL, Ed D (2017). Official American Thoracic Society/Infectious Diseases Society of America/Centers for Disease Control and Prevention clinical practice guidelines: diagnosis of tuberculosis in adults and children. Clin Infect Dais.

[17] Liu QY, Yu JT, Miao D, Ma XY, Wang HF, Wang W (2013). An exploratory study on STX6, MOBP, MAPT, and EIF2AK3 and late-onset Alzheimer's disease. Neurobiol Aging.

[18] Shaun P, Benjamin N, Kathe TB, Lori T, Manuel AR, David B (2007). PLINK: a tool set for whole-genome association and population-based linkage analyses. Am J Hum Genet.

[19] Goncalo R, Abecasis A, Auton L, Brooks D, Mark A, Pristo D (2012). An integrated map of genetic variation from 1,092 human genomes. Nature..

[20] Ward LD, Kellis M (2012). HaploReg: a resource for exploring chromatin states, conservation, and regulatory motif alterations within sets of genetically linked variants. Nucleic Acids Res.

[21] Carithers LJ, Ardlie K, Barcus M (2015). The genotype-tissue expression (GTEx) project. Biopreserv Biobank.

[22] Miao YR, Liu W, Zhang Q, Guo AY (2018). lncRNASNP2: an updated database of functional SNPs and mutations in human and mouse lncRNAs. Nucleic Acids Res.

[23] Lin J, Jiajia S, Liu D, Tangyuheng L, Wu T (2020). NCF2A Novel Genetic Variation in , the Core Component of NADPH Oxidase, Contributes to the Susceptibility of Tuberculosis in Western Chinese Han Population. DNA Cell Biol.

[24] ENCODE Project Consortium (2012). An integrated encyclopedia of DNA elements in the human genome. Nature..

[25] Ishikawa E, Ishikawa T, Morita YS, Toyonaga K, Yamada H, Takeuchi O, Kinoshita T, Akira S, Yoshikai Y, Yamasaki S (2009). Direct recognition of the mycobacterial glycolipid, trehalose dimycolate, by C-type lectin Mincle. J Exp Med.

[26] Schurz H, Kinnear CJ, Gignoux C, Wojcik G, van Helden PD, Gerard T (2019). A Sex-Stratified Genome-Wide Association Study of Tuberculosis Using a Multi-Ethnic Genotyping Array. Front Genet.

[27] Lin CJ, Lee SW, Liu CW, Chuu CP, Kao YH, Wu LSH (2019). Polymorphisms of suppressor of cytokine signaling-3 associated with susceptibility to tuberculosis among Han Taiwanese. Cytokine..

[28] Adeela S, Sobia R, Saqib M, Shahid S (2015). Role of leptin G-2548A polymorphism in age- and gender-specific development of obesity. J Biosci.

[29] Wang JY, Tsai CH, Lee YL, Lee LN, Hsu CL, Chang HC (2015). Gender-Dimorphic Impact of PXR Genotype and Haplotype on Hepatotoxicity During Antituberculosis Treatment. Medicine (Baltimore).

[30] Jiang L, Huang CL, Sun Q, Sun H, Guo T, Cheng Z (2015). The 5 '-UTR intron of the midgut-specific BmAPN4 gene affects the level and location of expression in transgenic silkworms. Insect Biochem Mol Biol.

[31] Jo BS, Choi SS (2015). Introns: the functional benefits of introns in genomes. Genomics..

[32] Santilli G, Thrasher AJ (2017). A new chapter on targeted gene insertion for X-CGD: do not skip the intron. Mol Ther.

[33] Sharma A, Steichen AL, Jondle CN, Mishra BB, Sharma J (2014). Protective role of Mincle in bacterial pneumonia by regulation of neutrophil mediated phagocytosis and extracellular trap formation. J Infect Dis.

[34] Behler-Janbeck F, Takano T, Maus R, Jennifer S, Ulrich AM (2016). C-type lectin Mincle recognizes Glucosyl-diacylglycerol of Streptococcus pneumoniae and plays a protective role in pneumococcal pneumonia. PLoS Pathog.

[35] van Rensburg IC, du Toit L, Walzl G, du Plessis N, Loxton AG (2018). Decreased neutrophil-associated miRNA and increased B-cell associated miRNA expression during tuberculosis. Gene..

[36] Lerman MSR, Leibowitz-Amit R, Sidi Y, Avni D (2014). The crosstalk between IL-22 signaling and miR-197 in human keratinocytes. PLoS One.

[37] Pahari S, Negi S, Aqdas M, Arnett E, Schlesinger LS, Agrewala JN (2020). Mycobacterium tuberculosisInduction of autophagy through CLEC4E in combination with TLR4: an innovative strategy to restrict the survival of. Autophagy..

[38] Dhiman R, Venkatasubramanian S, Paidipally P, Barnes PF, Tvinnereim A, Vankayalapati R (2014). Interleukin 22 inhibits intracellular growth of mycobacterium tuberculosis by enhancing calgranulin a expression. J Infect Dis.

[39] Alabbas SY, Begun J, Florin TH, Oancea L (2018). The role of IL-22 in the resolution of sterile and nonsterile inflammation. Clin Transl Immunol.

