# Supplementary material for: A case-control study on correlation between the single nucleotide polymorphism of CLEC4E and the susceptibility to tuberculosis among Han people in Western China
Source: BMC Infect Dis. 2021 Aug 10;21:788. doi: 10.1186/s12879-021-06448-2 (PMC8353747; doi:10.1186/s12879-021-06448-2)
Supplement: Supplementary file 1 — Additional file 1.Table S1. Allele and genotype distributions of CLEC4E SNPs polymorphisms in the TB group and healthy controls. Table S2. Comparison of 4 SNPs in relation to TB risk in the Chinese Han population. Table S3. Comparison of 4 SNPs polymorphisms in relation to TB risk in Western Chinese Han population stratified by age. Table S4. Comparison of 4 SNPs in relation to TB risk in the Chinese Han population stratified. Table S5. Functional annotation of rs10841856 and its closely linked SNPs (r2>0.90) using data from the Encyclopedia of DNA Elements Project. Table S6. Analyses of expression quantitative trait locus (eQTL) in rs10841856 and its closely linked SNPs (r2>0.90). Supplementary file 1. STROBE Statement—Checklist of items that should be included in reports of case-control studies. [file 12879_2021_6448_MOESM1_ESM.docx]

**Table S1.** Allele and genotype distributions of *CLEC4E* SNPs polymorphisms in the TB group and healthy controls

| SNP | Allele | Case n(%) | Control n(%) | OR (95% CI) ** | *P** | *P*** | *P**** | Genotype | Case n(%) | Control n(%) | *P** | *P**** |
| --- | --- | --- | --- | --- | --- | --- | --- | --- | --- | --- | --- | --- |
| rs10841856 | G | 814(45.22) | 1280(41.75) | 1.153(1.024,1.298) | **0.017** | **0.019** | 0.069 | GG | 176(19.56) | 265(17.29) | **0.025** | 0.100 |
| A>G | A | 986(54.78) | 1786(58.25) |  |  |  |  | GA | 462(51.33) | 750(48.92) | **0.040** | 0.160 |
|  |  |  |  |  |  |  |  | AA | 262(29.11) | 518(33.79) |  |  |
| rs10770847 | G | 558(31.03) | 888(29.00) | 1.091(0.960,1.241) | 0.134 | 0.183 | 0.536 | GG | 82(9.12) | 125(8.17) | 0.257 | 1.000 |
| A>G | A | 1240(68.97) | 2174(71.00) |  |  |  |  | GA | 394(43.83) | 638(41.67) | 0.194 | 0.776 |
|  |  |  |  |  |  |  |  | AA | 423(47.05) | 768(50.16) |  |  |
| rs10770855 | A | 783(43.55) | 1286(42.05) | 1.065.946,1.200) | 0.309 | 0.298 | 1.000 | AA | 166(18.46) | 260(17.00) | 0.295 | 1.000 |
| G>A | G | 1015(56.45) | 1772(57.95) |  |  |  |  | AG | 451(50.17) | 766(50.10) | 0.607 | 1.000 |
|  |  |  |  |  |  |  |  | GG | 282(31.37) | 503(32.90) |  |  |
| rs4480590 | A | 447(24.89) | 773 (25.31) | 0.976(0.852,1.119) | 0.743 | 0.730 | 1.000 | AA | 60(6.68) | 91(5.96) | 0.622 | 1.000 |
| G>A | G | 1349 (75.11) | 2281(74.69) |  |  |  |  | GA | 327(36.41) | 591(38.70) | 0.317 | 1.000 |
|  |  |  |  |  |  |  |  | GG | 511(56.90) | 845(55.34) |  |  |

SNP = single-nucleotide polymorphism;

*P** was calculated by Chi-square test;

*P*** and OR (95% CI) ** was adjusted by age and sex;

*P **** was calculated after Bonferroni correction;

OR = odds ratio; CI = confidence interval.

**Table S2.** Comparison of 4 SNPs in relation to TB risk in the Chinese Han population (Dominant and Recessive model)

| SNP | Dominant Model | | | | Recessive Model | | | |
| --- | --- | --- | --- | --- | --- | --- | --- | --- |
|  | OR (95% CI) ** | *P** | *P*** | *P**** | OR (95% CI) ** | *P** | *P*** | *P**** |
| rs10841856 | 1.226(1.024,1.469) | **0.017** | **0.027** | 0.068 | 1.189(0.959,1.473) | 0.161 | 0.114 | 0.644 |
| rs10770847 | 1.111(0.940,1.313) | 0.139 | 0.218 | 0.555 | 1.142(0.850,1.536) | 0.415 | 0.378 | 1.000 |
| rs10770855 | 1.074(0.898,1.284) | 0.437 | 0.437 | 1.000 | 1.112(0.895,1.383) | 0.361 | 0.339 | 1.000 |
| rs4480590 | 0.936(0.791,1.108) | 0.453 | 0.442 | 1.000 | 1.131(0.803,1.591) | 0.478 | 0.481 | 1.000 |

SNP = single-nucleotide polymorphism;

*P** was calculated by Chi-square test;

*P*** and OR (95% CI) ** was adjusted by age and sex;

*P **** was calculated after Bonferroni correction;

OR = odds ratio; CI = confidence interval.

**Table S3.** Comparison of 4 SNPs polymorphisms in relation to TB risk in Western Chinese Han population stratified by age

| SNP /Age | Allele | Case n(%) | Control n(%) | OR (95% CI) ^S^ | *P** | *P*^S^ | *P**** | Genotype | Case n(%) | Control n(%) | *P** | *P**** |
| --- | --- | --- | --- | --- | --- | --- | --- | --- | --- | --- | --- | --- |
| rs1084185＜40 | G | 381(45.68) | 779(42.25) | 1.146(0.972,1.351) | 0.096 | 0.105 | 0.385 | GG | 84(20.14) | 165(17.90) | 0.119 | 0.476 |
| A>G | A | 453(54.32) | 1065 (57.75) |  |  |  |  | AG | 213(51.08) | 449(48.70) | 0.148 | 0.592 |
|  |  |  |  |  |  |  |  | AA | 120(28.78) | 308(33.41) |  |  |
| rs10841856≥40 | G | 433(44.82) | 501(41.00) | 1.169(0.986,1.386) | 0.072 | - | 0.290 | GG | 92(19.05) | 100(16.37) | 0.088 | 0.352 |
| A>G | A | 533(55.18) | 721(59.00) |  |  |  |  | AG | 249(51.55) | 301(49.36) | 0.145 | 0.580 |
|  |  |  |  |  |  |  |  | AA | 142(29.40) | 210(34.37) |  |  |
| rs10770847＜40 | G | 260(31.18) | 533(29.00) | 1.107(0.927,1.323) | 0.254 | 0.263 | 1.000 | GG | 39(9.35) | 75(8.16) | 0.349 | 1.000 |
| A>G | A | 574(68.82) | 1305(71.00) |  |  |  |  | AG | 182(43.65) | 383(41.68) | 0.370 | 1.000 |
|  |  |  |  |  |  |  |  | AA | 196(47.00) | 461(50.16) |  |  |
| rs10770847≥40 | G | 298(30.91) | 355(29.00) | 1.095(0.911,1.317) | 0.333 | - | 1.000 | GG | 43(8.92) | 50(8.17) | 0.503 | 1.000 |
| A>G | A | 666(69.09) | 869(71.00) |  |  |  |  | AG | 212(43.98) | 255(41.67) | 0.358 | 1.000 |
|  |  |  |  |  |  |  |  | AA | 227(47.10) | 307(50.16) |  |  |
| rs10770855＜40 | A | 367(44.00) | 779(42.43) | 1.069(0.905,1.260) | 0.446 | 0.434 | 1.000 | AA | 79(18.94) | 154(16.78) | 0.380 | 1.000 |
| G>A | G | 467(56.00) | 1057(57.57) |  |  |  |  | AG | 209(50.12) | 471(51.31) | 0.954 | 1.000 |
|  |  |  |  |  |  |  |  | GG | 129(30.94) | 293(31.92) |  |  |
| rs10770855≥40 | A | 416(43.15) | 507（41.49） | 1.071（0.902,1.270） | 0.434 | - | 1.000 | AA | 87(18.05) | 106(17.35) | 0.507 | 1.000 |
| G>A | G | 548（56.85） | 715（58.51） |  |  |  |  | AG | 242(50.21) | 295(48.28) | 0.387 | 1.000 |
|  |  |  |  |  |  |  |  | GG | 153(31.74) | 210(34.37) |  |  |
| rs4480590 ＜40 | A | 214（25.78） | 519(25.03) | 1.047(0.867,1.264) | 0.678 | 0.631 | 1.000 | AA | 32(7.71) | 50(5.45) | 0.163 | 0.652 |
| G>A | G | 616(74.22) | 1375(74.97) |  |  |  |  | GA | 150(36.14) | 359(39.15) | 0.457 | 1.000 |
|  |  |  |  |  |  |  |  | GG | 233(25.40) | 508(55.40) |  |  |
| rs4480590 ≥40 | A | 233(24.12) | 314(25.74) | 0.917(0.754,1.115) | 0.386 | - | 1.000 | AA | 28(5.80) | 41(6.72) | 0.464 | 1.000 |
| G>A | G | 733(75.88) | 906(74.26) |  |  |  |  | GA | 177(36.65) | 232(38.03) | 0.543 | 1.000 |
|  |  |  |  |  |  |  |  | GG | 278(57.56) | 337(55.25) |  |  |

*P** was calculated by Chi-square test.

*P*^S^and OR (95% CI) ^S^were adjusted by age.

*P* ******* was calculated after Bonferroni correction.

SNP = single-nucleotide polymorphism; OR = odds ratio; CI = confidence interval.

**Table S4.** Comparison of 4 SNPs in relation to TB risk in the Chinese Han population stratified by age (Dominant and Recessive model)

| SNP/Age |  | Dominant Model | | | |  | Recessive Model | | | |
| --- | --- | --- | --- | --- | --- | --- | --- | --- | --- | --- |
|  | OR (95% CI) ^S^ | | *P** | *P*^S^ | *P**** | OR (95% CI) ^S^ | | *P** | *P*^S^ | *P**** |
| rs10841856 ＜40 | 1.232(0.957,1.587) | | 0.093 | 0.105 | 0.372 | 1.157(0.863,1.551) | | 0.328 | 0.329 | 1.000 |
| rs10841856 ≥40 | 1.258(0.972,1.627) | | 0.081 | - | 0.323 | 1.202(0.880,1.643) | | 0.247 | - | 1.000 |
| rs10770847 ＜40 | 1.130(0896,1.425) | | 0.284 | 0.304 | 1.000 | 1.169(0.778,1.754) | | 0.470 | 0.453 | 1.000 |
| rs10770847 ≥40 | 1.131(0.890,1.437) | | 0.314 | - | 1.000 | 1.101(0.719,1.686) | | 0.658 | - | 1.000 |
| rs10770855 ＜40 | 1.047(0.816,1.345) | | 0.721 | 0.717 | 1.000 | 1.166(0.863,1.574) | | 0.333 | 0.317 | 1.000 |
| rs10770855 ≥40 | 1.126(0.873,1.452) | | 0.360 | - | 1.000 | 1.049(0.768,1.434) | | 0.763 | - | 1.000 |
| rs4480590 ＜40 | 0.976(0.772,1.233) | | 0.800 | 0.839 | 1.000 | 1.472(0.928,2.333) | | 0.114 | 0.100 | 1.000 |
| rs4480590 ≥40 | 0.910(0.715,1.158) | | 0.444 | - | 1.000 | 0.854(0.520,1.403) | | 0.533 | - | 1.000 |

*P**was calculated by Chi-square test.

*P*^S^ and OR (95% CI) ^S^ were adjusted by sex.

*P**** was calculated after Bonferroni correction.

SNP = single-nucleotide polymorphism; OR = odds ratio; CI = confidence interval.

**Table S5.** Functional annotation of rs10841856 and its closely linked SNPs (r^2^>0.90) using data from the Encyclopedia of DNA Elements Project

| SNP | r^2^ | Annotation | Promoter histone makers† | Enhancer histone marks‡ | DNAse§ | Proteins bound¶ | Motifs changed\|\| |
| --- | --- | --- | --- | --- | --- | --- | --- |
| rs10841847 | 0.95 | intronic |  |  | THYM |  | 4 altered motifs |
| rs7307228 | 0.95 | intronic |  | BLD |  |  | 10 altered motifs |
| rs4242896 | 0.96 | intronic |  | BLD |  |  |  |
| rs6487242 | 0.96 | intronic |  |  |  |  | 4 altered motifs |
| rs7139227 | 0.92 | intronic |  | BLD | BLD | USF1 | CTCF |
| rs4562874 | 1 | intronic |  |  |  |  | Pou2f2 |
| rs10841856 | 0.97 | intronic |  |  | BLD |  | 20altered motifs |
| rs11046135 | 0.96 | intergenic | BLOOD | BLD, FAT | 10 tissues |  | Foxp1,Pou2f2 |
| rs7485954 | 0.94 | intergenic |  | BLD |  |  | 11 altered motifs |

†Evidence of local H3K4Me1 and H3K27Ac modification (cell lines/types).

‡Evidence of local H3K4Me3 modification (cell lines/types).

§Evidence of chromatin hypersensitivity to DNase (cell lines/types: if >3, only the number is included).

¶ChIP-seq experiments indicate an alteration in the binding of transcription factor.

||Evidence of alteration in the regulatory motif (if >3, only the number is included).

**Table S6.** Analyses of expression quantitative trait locus (eQTL) in rs10841856 and its closely linked SNPs (r^2^>0.90)

| Variant | Gene | *P* | Effect Size | Tissue |
| --- | --- | --- | --- | --- |
| rs10841847 | *CLEC4E* | 7.00×10^-16^ | -0.16 | Whole Blood |
|  | *RP11-561P12.5* | 6.30×10^-20^ | 0.31 | Whole Blood |
| rs73027228 | *CLEC4E* | 5.60E-16 | -0.16 | Whole Blood |
|  | *RP11-561P12.5* | 5.70E-20 | 0.31 | Whole Blood |
| rs4242896 | *CLEC4E* | 6.00E-18 | -0.17 | Whole Blood |
|  | *RP11-561P12.5* | 1.40E-20 | 0.31 | Whole Blood |
| rs6487242 | *CLEC4E* | 4.00E-18 | -0.17 | Whole Blood |
|  | *RP11-561P12.5* | 1.40E-20 | 0.31 | Whole Blood |
| rs7139227 | *CLEC4E* | 4.50E-16 | -0.16 | Whole Blood |
|  | *RP11-561P12.5* | 9.50E-20 | 0.31 | Whole Blood |
| rs4562874 | *CLEC4E* | 1.30E-17 | -0.17 | Whole Blood |
|  | *RP11-561P12.5* | 2.90E-21 | 0.32 | Whole Blood |
| rs10841856 | CLEC4E | 2.20E-18 | -0.17 | Whole Blood |
|  | RP11-561P12.5 | 4.30E-20 | 0.3 | Whole Blood |
| rs11046135 | *CLEC4E* | 2.00E-18 | -0.17 | Whole Blood |
|  | *RP11-561P12.5* | 1.50E-20 | 0.31 | Whole Blood |
| rs7485954 | *CLEC4E* | 3.80E-18 | -0.17 | Whole Blood |
|  | *RP11-561P12.5* | 3.40E-20 | 0.31 | Whole Blood |

Data source: Genotype-Tissue Expression (GTEx) Project. Only when the P＜10^-15^ is included.

Supplementary file 1

STROBE Statement—Checklist of items that should be included in reports of ***case-control studies***

|  | | **Item No** | **Recommendation** |
| --- | --- | --- | --- |
| **Title and abstract** | | 1 | (*a*) Indicate the study’s design with a commonly used term in the title or the abstract |
|  |  |  | (*b*) Provide in the abstract an informative and balanced summary of what was done and what was found |
| **Introduction** | | | |
| Background/rationale | | 2 | Explain the scientific background and rationale for the investigation being reported |
| Objectives | | 3 | State specific objectives, including any prespecified hypotheses |
| **Methods** | | | |
| Study design | | 4 | Present key elements of study design early in the paper |
| Setting | | 5 | Describe the setting, locations, and relevant dates, including periods of recruitment, exposure, follow-up, and data collection |
| Participants | | 6 | (*a*) Give the eligibility criteria, and the sources and methods of case ascertainment and control selection. Give the rationale for the choice of cases and controls |
|  |  |  | (*b*) For matched studies, give matching criteria and the number of controls per case |
| Variables | | 7 | Clearly define all outcomes, exposures, predictors, potential confounders, and effect modifiers. Give diagnostic criteria, if applicable |
| Data sources/ measurement | | 8* | For each variable of interest, give sources of data and details of methods of assessment (measurement). Describe comparability of assessment methods if there is more than one group |
| Bias | | 9 | Describe any efforts to address potential sources of bias |
| Study size | | 10 | Explain how the study size was arrived at |
| Quantitative variables | | 11 | Explain how quantitative variables were handled in the analyses. If applicable, describe which groupings were chosen and why |
| Statistical methods | | 12 | (*a*) Describe all statistical methods, including those used to control for confounding |
|  |  |  | (*b*) Describe any methods used to examine subgroups and interactions |
|  |  |  | (*c*) Explain how missing data were addressed |
|  |  |  | (*d*) If applicable, explain how matching of cases and controls was addressed |
|  |  |  | (*e*) Describe any sensitivity analyses |
| **Results** | | | |
| Participants | | 13* | (a) Report numbers of individuals at each stage of study—eg numbers potentially eligible, examined for eligibility, confirmed eligible, included in the study, completing follow-up, and analysed |
|  |  |  | (b) Give reasons for non-participation at each stage |
|  |  |  | (c) Consider use of a flow diagram |
| Descriptive data | | 14* | (a) Give characteristics of study participants (eg demographic, clinical, social) and information on exposures and potential confounders |
|  |  |  | (b) Indicate number of participants with missing data for each variable of interest |
| Outcome data | | 15* | Report numbers in each exposure category, or summary measures of exposure |
| Main results | | 16 | (*a*) Give unadjusted estimates and, if applicable, confounder-adjusted estimates and their precision (eg, 95% confidence interval). Make clear which confounders were adjusted for and why they were included |
|  |  |  | (*b*) Report category boundaries when continuous variables were categorized |
|  |  |  | (*c*) If relevant, consider translating estimates of relative risk into absolute risk for a meaningful time period |
| Other analyses | 17 | Report other analyses done—eg analyses of subgroups and interactions, and sensitivity analyses | |
| **Discussion** | | | |
| Key results | 18 | Summarise key results with reference to study objectives | |
| Limitations | 19 | Discuss limitations of the study, taking into account sources of potential bias or imprecision. Discuss both direction and magnitude of any potential bias | |
| Interpretation | 20 | Give a cautious overall interpretation of results considering objectives, limitations, multiplicity of analyses, results from similar studies, and other relevant evidence | |
| Generalisability | 21 | Discuss the generalisability (external validity) of the study results | |
| **Other information** | | | |
| Funding | 22 | Give the source of funding and the role of the funders for the present study and, if applicable, for the original study on which the present article is based | |

*Give information separately for cases and controls.

Our study is a retrospective case-control study. We have checked the contents of the manuscript one by one according to Checklist and can confirm that this manuscript contains all the required items.
